# Site-specific hydrogen isotope measurements of vanillin by ^2^H-qNMR and GC-IRMS

**DOI:** 10.1007/s00216-025-05920-1

**Published:** 2025-06-06

**Authors:** Phuong Mai Le, Markus Greule, Stanislav Sokolenko, Serge Akoka, Gérald Remaud, Peter Costa, Kathy Sharon Isaac, Andre Simpson, Frank Keppler, Juris Meija

**Affiliations:** 1 Metrology Research Centre, National Research Council Canada, 1200 Montreal Road, Ottawa, ON K1A-0R6 Canada; 2 Institute of Earth Sciences, Heidelberg University, Im Neuenheimer Feld 236, Heidelberg, 69120 Germany; 3 Department of Process Engineering and Applied Science, Dalhousie University, 5273 DaCosta Row, Halifax, NS B3H-4R2 Canada; 4 CEISAM, Nantes Université-CNRS, UMR6230, 2 rue de la Houssinière, Nantes, F-44000 France; 5 Environmental NMR Centre, University of Toronto, 1265 Military Trail, Toronto, ON M1S-1A4 Canada

**Keywords:** Site-specific hydrogen isotope ratio, ^2^H-qNMR, GC-IRMS, Vanillin authentication

## Abstract

Stable isotope analyses are powerful techniques to detect counterfeiting of food products, especially in vanillin, one of the world’s most popular flavors used in food and pharmaceutical industries. Although a stable carbon isotopic ratio provides an invaluable new source of isotopic information, the analysis of the hydrogen site-specific (^2^H/^1^H) isotopic distribution in vanillin gives valuable complementary isotopic information allowing a complete isotopic fingerprint of the vanillin molecule for authentication. However, there are only a limited number of studies that compare the validity of the results obtained by IRMS and NMR. Here, we present site-specific isotope analysis (also known as position-specific isotope analysis, PSIA) of hydrogen isotopic measurements of vanillin by two independent measurement methods, ^2^H-qNMR and GC-IRMS, and compare the results obtained by these two methods. ^2^H-qNMR allows isotopic measurements of all hydrogen atoms (except for OH) of the vanillin molecule while GC-IRMS measures only the hydrogen atoms of the methoxy group (H5). ^2^H/^1^H values of the vanillin H5 from six vanillin demonstrate remarkable agreement between the two techniques with uncertainty well below 1%. Position-specific GC-IRMS on the methoxy group measurements provides three-fold smaller measurement uncertainties while requiring a considerably smaller sample size compared to ^2^H-qNMR. This quantitative hydrogen isotopic study extends our previous successful work comparing and validating the stable carbon isotopic of the vanillin methoxy group using ^13^C-qNMR and GC-IRMS. Compound-specific isotope analysis (CSIA) using GC-IRMS was also described in this work. Both qNMR and partial PSIA GC-IRMS can be considered as complementary analytical methods, and their combined use provides reliable results.

**Graphical Abstract:**

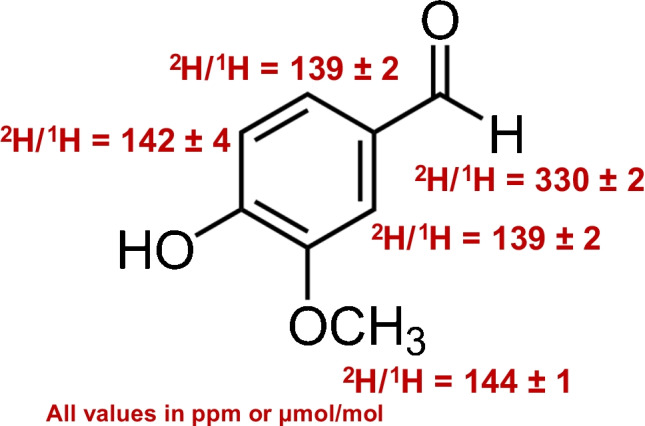

**Supplementary Information:**

The online version contains supplementary material available at 10.1007/s00216-025-05920-1.

## Introduction

The food industry today has to face increasingly sophisticated counterfeiting such as the use of synthetic ingredients when a natural origin is labeled. Consumers are increasingly interested in the authenticity and traceability of food products, making the authentication of food products of primary importance to consumers and industries alike. Stable isotope analyses are often used to trace the geographical origin of food and agricultural practices, or to detect differences in the production techniques or processing methods [1–3]. Isotope ratio mass spectrometry (IRMS) is the most commonly used technique to determine the bulk isotope ratio for a given molecule [2], whereas quantitative nuclear magnetic resonance spectrometry (qNMR) can provide the isotopic composition of an element at a specific molecular position [4]. The position-specific isotope analysis (PSIA, often termed “site-specific” in the literature) can provide additional information about the history of substances from raw materials to finished products. For example, ethanol derived from corn and agave both have similar bulk values of the ^13^C/^12^C isotope ratio. However, the ^13^C/^12^C isotope ratios in the –CH_2_– and –CH_3_ carbon atom differ significantly [5].

Vanillin is one of the world’s most popular flavors used in food and pharmaceutical industries. Owing to its scarcity and high production costs, naturally extracted vanillin makes a small fraction (less than 1%) of yearly produced vanillin, with most of the vanillin on the market being derived from guaiacol and lignin, the remaining part being ensured by biovanillin from several precursors [6, 7]. Compound-specific isotope analysis (CSIA, often referred to as bulk) of hydrogen and carbon using either elemental analyzer-RMS (EA-IRMS) or gas chromatography-IRMS (GC-IRMS) has both been used to distinguish vanillin from ex-beans and from two other important origins, guaiacol and lignin, and to authenticate vanilla species (*V. planifolia* v *V. tahitensis*) and their geographical origin [8–12]. CSIA using GC-IRMS has been proposed, either to determine the ^13^C composition of the four main analytes of vanilla extracts (vanillin, parahydroxy-benzaldehyde, vanillic acid, and parahydroxy-benzoic acid) or for working with little material as in final products (as ice cream) [8, 13, 14]. In addition, partial PSIA by GC-IRMS for isotopic characterization of vanillin’s methoxy group [15, 16] has prompted the exploration of position-specific measurements. Despite the high sensitivity of IRMS and GC-IRMS methods, none of these methods provides a full ^2^H and ^13^C ratio at individual positions (position-specific isotopic distribution) within a vanillin molecule, resulting in some limitations in the authentication of vanillin samples [15, 16].

Using ^13^C-qNMR, ^13^C/^12^C isotope ratios at all eight carbon positions within the vanillin molecule can be measured, allowing clear discrimination of the synthetic vanillin from guaiacol and lignin, and synthetic and biosynthetic vanillin from several natural precursors [17–19]. The reliability of ^13^C-qNMR site-specific carbon isotope measurements from vanillin has been addressed through inter-laboratory comparisons [20, 21] and by comparison with PSIA GC-IRMS [22], supporting the production of carbon isotopic certified reference materials of vanillin [23]. The use of ^2^H-qNMR measuring ^2^H/^1^H ratios has been successfully established in food authentication studies with applications in provenance studies of wine, spirits, and other beverages [3, 24]; detection of sugar addition in wines [25–27]; and provenance of vinegar [28]. This approach was also used to track the sources [29] or metabolic pathway of compounds in environmental chemistry [30, 31] and human physiology [32]. The advantages of ^2^H/^1^H analysis by qNMR analysis are as follows: (i) deuterium exists in all organic molecules as an isotopic probe; (ii) the ^2^H chemical shifts are generally identical to the corresponding ^1^H chemical shifts; (iii) due to the large variation of deuterium composition compared to the scale of carbon isotope at natural abundance [33, 34], the target precision of a few percent (%) for ^2^H-qNMR is more easily reached than that required for ^13^C-qNMR (a few permille, ‰) [4]. The main disadvantages of working with ^2^H are the possible chemical exchange (for example position 4 of vanillin is exchangeable in boiling water [35]) and the much lower sensitivity [4, 33]. Site-specific measurements of ^2^H/^1^H by ^2^H-qNMR have proved to be an efficient way to authenticate natural vanillin extracts [36], enabling the detection of up to 10% of synthetic or semi-synthetic vanillin [35] and the combination of ^2^H and ^13^C isotopic measurements provides strong evidence to authenticate vanillin produced from the different precursors [36]. Although the use of ^2^H-qNMR is an established method for vanillin authentication, a limited number of studies have been devoted to compare this method with other approaches.

In this context, the primary objective of this study is to compare ^2^H-qNMR and GC-IRMS for measuring the site-specific hydrogen isotopic composition in various vanillin samples.

## Material and methods

### Chemicals

Dimethyl sulfoxide-*d*_6_ (DMSO-*d*_6_, ^1^H-NMR solvent) was obtained from Cambridge Isotope Laboratories (Andover, MA, USA). Acetonitrile (CH_3_CN, ^2^H-NMR solvent), hexafluorobenzene (C_6_F_6_, NMR frequency lock), and trifluoroacetic acid (TFA, used to shift the vanillin –OH signal and to avoid overlaps in ^2^H-NMR spectra) were all purchased from Sigma-Aldrich (Oakville, ON, Canada). Maleic acid (SRM grade, TraceCERT®, having a chemical purity of 0.9989 ± 0.0031 g/g) was used as qNMR internal standard for purity determination and was obtained from Fluka. Three sealed NMR tubes containing TMU, C_6_F_6_, and ethanol (ERM-AE200 a, b, c) were obtained from European Reference Materials (ERM, Geel, Belgium). Isotopic reference material of tetramethylurea (TMU, ERM-AE003) with ^2^H/^1^H value of 123 ± 0.35 ppm from ERM was used as calibrator for ^2^H-qNMR. All chemicals are pure (min. 99% purity). NMR tubes (5 mm and 10 mm inner diameter) were obtained from Wilmad LabGlass (Buena, NJ, USA).

The following vanillin samples used in this study are commercial products obtained from different suppliers. Both VANA-1 and VANB-1 are vanillin carbon isotopic certified reference materials (CRMs) from National Research Council Canada (NRC), Ottawa, Canada [23]. Also, five more vanillin samples were used: one vanillin from Alfa Aesar, identified as VAN-1, with product number A11169 (product of USA); three from Sigma-Aldrich, identified as VAN-4, VAN-8, and VAN-K, with product numbers 94752-100 g, W310700-sample-K, and W310727-sample-K, respectively (the first two are products from China, and the third one from Germany). Lastly, a vanillin sample from Haarmann & Reimer (H & R^*c*^, product of Germany) which is identified as VAN-HR. The chemical purity of vanillin in each vanillin material was determined at the NRC by the quantitative ^1^H-NMR (^1^H-qNMR) using the general method described previously [37]. The purity estimates of vanillin, expressed in g/g, are 0.9962 ± 0.024 (VANA-1), 0.9954 ± 0.023 (VANB-1), 0.9856 ± 0.039 (VAN-1), 0.9931 ± 0.032 (VAN-4), 0.9932 ± 0.037 (VAN-8), 0.9898 ± 0.030 (VAN-HR), and 0.9695 ± 0.033 (VAN-K).

### IRMS analyses

#### Bulk hydrogen isotope measurements of vanillin

Each vanillin sample (3.5 mg) was dissolved in 1 mL ethyl acetate and 0.4 µL of the solution was injected into the GC-TC-IRMS system using a 10-µL syringe for liquid injections (Hamilton, SYR 10 µL, 701 N). The H_3_^+^-factor was determined to be 2.88 ppm/nA. All *δ*(^2^H) bulk values were calibrated using a two-point linear interpolation with two in-house vanillin reference materials. The hydrogen isotope delta values of these materials relative to Vienna Standard Mean Ocean Water (VSMOW) are *δ*_VSMOW_(^2^H) = 79.7 ± 1.2‰ (Vanillin Fluka) and *δ*_VSMOW_(^2^H) = −155.0 ± 1.5‰ (Eurovanillin supreme) [38]. Twenty measurements were analyzed for each in-house vanillin reference, and four measurements for each vanillin sample were performed.

#### Generation of iodomethane from vanillin for GC-IRMS analysis

Analysis of *δ*(^2^H) from CH_3_I released upon treatment of the vanillin samples with aqueous hydriodic acid (Acros, Thermo Fisher Scientific, Geel, Belgium) was carried out using the method described by Greule et al. [38]. In short, hydriodic acid (0.25 mL) was added to the vanillin sample (5 mg) in a crimp-top glass vial (1.5 mL; IVA Analysentechnik, Meerbusch, Germany). The vials were sealed with crimp caps containing PTFE-lined butyl rubber septa (thickness 1.0 mm) and incubated at 130 °C for 30 min. Afterwards, samples were allowed to equilibrate at room temperature (22 ± 0.5 °C) for at least 30 min before 30 µL of the headspace was directly injected into the GC using a 100 μL gas-tight syringe (SGE Analytical Science).

#### Isotope analysis of methoxy groups using GC-IRMS

Hydrogen isotope delta values of CH_3_I were measured using an HP 6890 N gas chromatograph (Agilent, Santa Clara, USA) equipped with an auto sampler A200S (CTC Analytics, Zwingen, Switzerland), coupled to a MAT253 isotope ratio mass spectrometer (Thermo Fisher Scientific, Bremen, Germany) via a thermo conversion reactor (Al_2_O_3_ ceramic tube, 320 mm length, 0.5 mm i.d., reactor temperature 1450 °C) and a GC Combustion III Interface (Thermo Quest Finnigan, Bremen, Germany). The GC was fitted with a Zebron ZB-5MS capillary column (Phenomenex, Torrance, USA) (30 m × 0.25 mm i.d., df = 1 μm) and the following GC conditions were employed: split injection (4:1), initial oven temperature at 40 °C for 3.8 min, ramp at 50 °C/min to 110 °C. High-purity helium (5 N) was used as a carrier gas at a constant flow of 0.7 mL/min. A tank of high-purity hydrogen gas (Alphagaz™2H2, Air Liquide, Düsseldorf, Germany) was used as the monitoring gas. The H_3_^+^-factor was 2.83 ± 0.01 ppm/nA during the measurement period. All hydrogen isotope delta values were calibrated with a linear calibration curve using reference materials HUBG2 (potassium methyl sulfate) and HUBG3 (birch wood) [39, 40]. Both were measured by EA-IRMS and calibrated to the VSMOW scale using the reference materials VSMOW2 (0 ± 0.3‰) and SLAP2 (−427.5 ± 0.3‰). The calibrated values for HUBG2 and HUBG3 are −102.0 ± 1.3‰ (*n* = 32) and −272.9 ± 1.5‰ (*n* = 11) relative to the VSMOW [39, 40].

### NMR analyses

#### ^2^H-qNMR for ^2^H isotopic measurements

The ^2^H-qNMR spectra were acquired using a Bruker Avance III 500 spectrometer equipped with a “deuterium” 10 mm o.d. NMR-BBFO probe having a proton decoupling channel, field-frequency stabilization (lock) channel at ^19^F frequency, and an actively shielded z-gradient. The temperature of the probe was set to 308 K. Probe tuning and matching were performed at the recording frequency of 76.79 MHz. Samples were automatically shimmed using the ^19^F lock channel, followed by manual verification to check the obtained results. A broadband ^1^H composite pulse decoupling (Waltz-16) was applied continuously during ^2^H-qNMR acquisition.

#### NMR spectrometer qualification

**Fig. 1:**
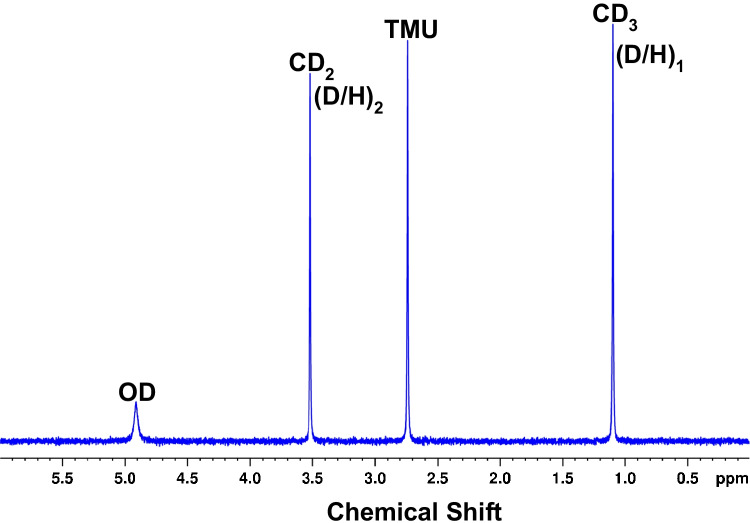
^2^H-qNMR spectrum of ethanol and TMU mixture

Three sealed NMR tubes containing TMU, C_6_H_6_, and EtOH certified reference materials were used to evaluate the NMR performance. The ^2^H-NMR spectra were recorded using the procedure 90° pulse (spectral width 15.6 ppm (1200 Hz), a 6.82s acquisition time, a relaxation delay of 1.1 s with the 8.0 s repetition time, 8 dummy scans, and 256 scans), resulting in the overall acquisition time of 65 min per spectra. The TMU peak at 2.73 ppm was used as a chemical shift standard and for quantitative calculations. All spectra were transformed with line broadening (LB) of 0 and manually phased. Ten spectra were consecutively acquired for each ethanol sample with a typical ^2^H-qNMR spectrum of ethanol shown in Fig. 1.

#### ^2^H-qNMR measurements of vanillin samples

The sample preparation and ^2^H-qNMR measurements followed the AOAC method 2006.05 [41]. Each sample was gravimetrically prepared by combining 2.1 g of vanillin, 0.44 mL (0.41 g) of TMU, 3.1 mL (2.455 g) of acetonitrile, 0.20 mL (0.325 g) of C_6_F_6_, and 0.1 mL (0.149 g) of TFA (to displace the OH signal). The vial was then carefully closed with the cap and vortex-mixed until a clear solution was obtained. The mixture was transferred into a 10 mm NMR tube which was flame-sealed to prevent evaporation of the solvent. The temperature of the probe and sample was set to 308 K. For most vanillin samples, the 90°-^2^H pulse was calibrated to 22.5 µs. The *T*_1_ values of ^2^H resonances were determined using an inverse recovery sequence (see Electronic supplementary material, Fig. S1). The acquisition conditions were as follows: the acquisition time (AQ) of 4 s, relaxation delay of 0.15 s, (the longest *T*_1_ of 0.688 s at TMU peak, see the Electronic supplementary material, Fig. S1) to ensure that the slowest decaying signal in the sample achieves quantitative relaxation of the magnetization, spectra width of 20 ppm, spectra width of 20 ppm, 8 dummy scans, and number of scans (NS) of 2048, resulting in the overall acquisition time of 143 min per spectrum. At least three spectra were acquired consecutively for each vanillin sample. All spectra were calibrated to the TMU internal standard reference.

#### Data processing and treatment of ^2^H-NMR signals

**Fig. 2:**
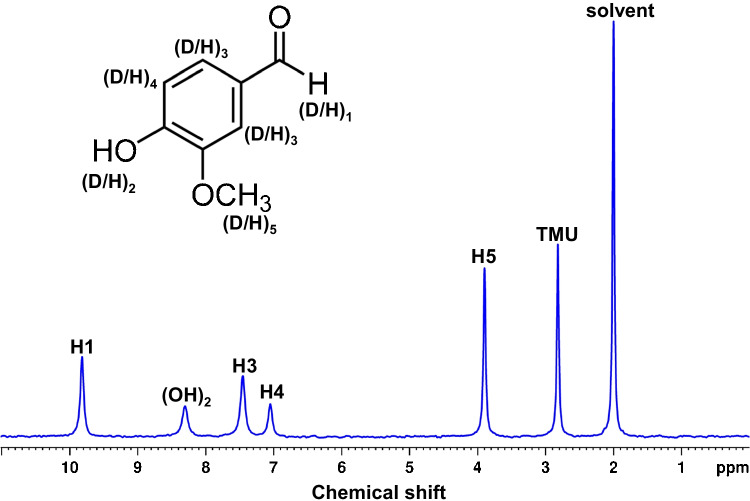
^2^H-qNMR spectrum of vanillin

Spectra were processed using the TopSpin software (v3.6) with the same parameters and the same procedure for free induction decays of 65 K: (i) an exponential multiplication inducing a 2 Hz line broadening, (ii) manual phasing, and (iii) automatic baseline correction using a third-order polynomial. A typical ^2^H-qNMR spectrum of vanillin is shown in Fig. 2.

The deuterium signal peak areas were obtained by peak fitting using the R package rnmrfit [42, 43]. The following integration windows were applied on the chemical shift scale: 3.80–3.40 ppm (EtOH, CH_2_); 3.00–2.60 ppm (TMU), 1.30–0.90 ppm (EtOH, CH_3_), 10.20–9.30 ppm (H1), 10.40–9.40 ppm (H1), 7.90–7.25 ppm (H3), 7.20–76.80 ppm (H4), 4.40–3.60 ppm (H5), and 3.00–2.60 ppm (TMU) for vanillin sample.

#### Site-specific hydrogen isotope values

The site-specific ^2^H/^1^H isotope ratios *R* (expressed in µmol/mol, commonly written as ppm) were measured by ^2^H-qNMR and calibrated against TMU isotopic reference material having the known ^2^H/^1^H value of 123 ± 0.35 ppm, *R*(^2^H/^1^H, TMU) [17, 41]:

1 $$R\left({}^2H/{}^1H,\;van-i\right)=R\left({}^2H/{}^1H,\;TMU\right)\;\left({}S_{van-i}/{}S_{TMU}\right)\;\left({}P_{TMU}/{}P_{van-i}\right)\;\left({}m_{TMU}/{}m_{van}\right)\;\left({}M_{van}/{}M_{TMU}\right)$$

Here, *S*_TMU_ and *S*_van-*i*_ are the peak areas of TMU and ^2^H signal from vanillin at any given position (site), respectively. *P*_TMU_ and *P*_van-*i*_ are the numbers of equivalent deuterium atoms corresponding to the TMU signal and each vanillin signal, *M* is the molar mass, and *m* is the mass used. The mass of vanillin in each sample was corrected to take into account its purity, i.e., *m*_van_ = *w*_van_*m*_sample_, where *w*_van_ is the chemical purity of vanillin in the given sample and *m*_sample_ is the amount of sample weighed (Electronic supplementary material, Table S1).

## Results and discussion

### Hydrogen isotope results by GC-IRMS

Hydrogen isotope delta values for bulk vanillin and for hydrogen of methoxy group at the position H5 (obtained by GC-IRMS) are expressed relative to VSMOW. While some authors prefer to use milliurey (mUr) as the unit for hydrogen isotope delta [44], we continue to use permille (‰) for comparison with previous works [15, 16] as shown in Table 1.

**Table 1:** Hydrogen isotope composition values for bulk vanillin, for vanillin methoxy group (H_5_), and their standard uncertainties (*n* = 4 and 5, respectively, except *n* = 10 for hydrogen of H_5_ of VANA-1 and VANB-1). All values reported in permilles (‰) relative to the VSMOW

Material	*δ*(^2^H, bulk)	*δ*(^2^H, H5_,_ methoxy group)
VANA-1	58.9 ± 2.3	−79.4 ± 1.7
VANB-1	37.7 ± 1.8	−56.4 ± 1.9
VAN-1	−5.6 ± 2.0	−90.2 ± 1.7
VAN-4	43.8 ± 2.0	−88.8 ± 1.8
VAN-8	28.6 ± 2.2	−41.2 ± 2.1
VAN-K	−151.5 ± 2.2	−180.1 ± 2.4
VAN-HR	−55.7 ± 2.8	−173.1 ± 2.8

The large discrepancy in the *δ*(^2^H, bulk) and *δ*(^2^H, H5, methoxy group) values of the different samples likely reflects the diverse origin of the vanillin samples. Most synthetic vanillin is produced from a petrochemical precursor, guaiacol [6, 8]. Indeed, five vanillin samples show bulk values of *δ*(^2^H), typical of synthetic vanillin from guaiacol, that is, from −25‰ to +117‰ [15, 45, 46]. The bulk values of *δ*(^2^H) for VAN-HR and VAN-K, on the other hand, are consistent with natural vanillin, which is characterized with *δ*(^2^H) ranging from −180‰ to −50‰ [16, 45, 46]. To some extent, the very large discrepancy between the *δ*(^2^H, bulk) and *δ*(^2^H, H5, methoxy group) values can also be attributed to different precursor substances and different (bio)synthesis pathways, which is also observable when measuring the ^13^C/^12^C isotope values of identical samples (Table S3). In the case of hydrogen isotope signatures, the sometimes large difference between bulk and methoxy groups can be explained by the biosynthesis of methoxy groups in the case of natural precursors. This is often accompanied by a strong fractionation of one hydrogen atom of the methoxy group formed, which leads to significantly more negative *δ*(^2^H, H5) values of the entire methoxy group [47].

### Hydrogen isotopic composition of vanillin by ^2^H-qNMR

#### Qualification of NMR spectrometer

The accuracy of ^2^H-qNMR results greatly depends on instrument hardware specifications. Therefore, prior to performing ^2^H-qNMR measurements on vanillin samples, the performance of the NMR was evaluated using reference materials with known isotopic composition. The qualification procedure was performed using TMU reference material with certified ^2^H/^1^H ratio, and three ethanol standards with certified ^2^H/^1^H ratios. The proton decoupling sequence (such as Waltz-16) can be used during the acquisition to simplify the spectral analysis of ^2^H-qNMR spectra [33]. With the 500 MHz NMR instrument (University of Toronto) using the above optimized experimental conditions, the instrument qualification procedure showed (i) half-width of methyl signal of TMU internal standard ≤ 0.5 Hz when the EtOH spectra were transformed without apodization and (ii) the total of 256 scans were found to be sufficient for achieving a good signal-to-noise ratio (> 200) with LB = 2 for methyl signal of ethanol samples. These parameters correspond to the resolution and sensitivity required for ^2^H-qNMR [35, 41]. Further, the repeatability standard deviation < 0.5% (*n* = 10) for both CH_3_ and CH_2_ groups (see Table 2) meets the precision requirements for ^2^H/^1^H measurements.

**Table 2:** Site-specific ^2^H/^1^H values and their standard uncertainties (*n*=10) for three reference materials of ethanol, measured by ^2^H-qNMR. Isotope ratios *R* are reported in ppm (µmol/mol)

CRM		*R*(^2^H/^1^H, CH_2_)	RSD	*R*(^2^H/^1^H, CH_3_)	RSD
EtOH-H	NRC ERM certified	129.88 ± 0.52 129.90 ± 0.15	0.36%	111.55 ± 0.69 110.47 ± 0.12	0.31%
EtOH-M	NRC ERM certified	130.14 ± 0.52 130.52 ± 0.13	0.40%	104.26 ± 0.69 103.33 ± 0.11	0.31%
EtOH-L	NRC ERM certified	125.52 ± 0.52 125.62 ± 0.35	0.49%	93.38 ± 0.69 92.60 ± 0.10	0.49%

Natural variations of the hydrogen isotopes are often expressed as isotope deltas relative to VSMOW. However, the absolute hydrogen isotope ratio in SMOW, *R*(^2^H/^1^H, SMOW) = 155.76 ± 0.05 ppm (µmol/mol) [48], permits a conversion between the isotope ratio values and isotope delta values.

As seen from Table 2, in all cases, the values measured at the NRC by ^2^H-qNMR agree to within their uncertainties with those certified by the ERM, indicating that our NMR spectrometer is suitably qualified to measure ^2^H/^1^H ratios in natural samples.

#### ^2^H-qNMR measurements of vanillin sample

The precision and trueness of ^2^H-qNMR measurements depends mainly on the S/N achievable in a reasonable time which in turn is a function of the acquisition time (AQ), the delay between pulses (repetition time), and are also intrinsically dependent on precise, accurate, and robust peak area detection [4]. The acquisition conditions were as follows: the AQ of 4 s, relaxation delay of 0.15 s, longest *T*_1_ = 0.688 s (see electronic supplementary material, Fig. S1) to recover at least 99.91% of the magnetization. The NS of 2048 was used and optimized to achieve a good signal-to-noise ratio (≥ 540) for the CH_2_D signal of acetonitrile at the chemical shift of 2 ppm. Furthermore, the high precision of ^2^H-qNMR depends on precision and robust peak area evaluation. To this effect, the deconvolution model, rnmrfit, has been developed to provide two-fold improvement in the peak area precision over commercially available tools [43]. This model fits Lorentz or Voigt peaks in both the real and imaginary dimensions simultaneously, ensuring more reliable quantification [42]. Using these optimized experimental conditions and deconvolution model, we have successfully measured the ^2^H/^1^H values of vanillin at the positions H1, H3, H4, and H5.

#### Site-specific isotope ratios in vanillin samples by ^2^H-qNMR

The ^2^H/^1^H values for vanillin samples obtained by ^2^H-qNMR are traceable to TMU reference and are shown in Table 3. Due to the limited quantity of VAN-HR sample, we focused on measuring the four-hydrogen site-specific composition of this vanillin by qNMR, and did not measure the hydrogen of methoxy group of this vanillin by GC-IRMS.

**Table 3:** Hydrogen isotope ratio values for vanillin samples and their standard uncertainties measured by ^2^H-qNMR and GC-IRMS (*n* = 3 and 5, respectively). All values reported in ppm (µmol/mol)

	By ^2^H-qNMR				By GC-IRMS
Material	*R*(^2^H/^1^H, H1)	*R*(^2^H/^1^H, H3)	*R*(^2^H/^1^H, H4)	*R*(^2^H/^1^H, H5)	*R*(^2^H/^1^H, H5)
VANA-1	330.04 ± 2.23	138.90 ± 1.42	142.38 ± 3.62	143.55 ± 0.86	143.40 ± 0.27
VANB-1	267.79 ± 2.19	139.35 ± 1.42	147.09 ± 3.62	146.34 ± 0.86	146.98 ± 0.30
VAN-1	258.53 ± 2.17	133.08 ± 1.41	140.93 ± 3.62	141.15 ± 0.85	141.72 ± 0.27
VAN-4	305.30 ± 2.21	142.79 ± 1.42	146.79 ± 3.62	142.01 ± 0.85	141.92 ± 0.29
VAN-8	254.13 ± 2.17	141.35 ± 1.42	148.64 ± 3.62	150.10 ± 0.86	149.35 ± 0.33
VAN-K	121.08 ± 2.10	122.38 ± 1.41	143.30 ± 3.62	126.51 ± 0.84	127.70 ± 0.37
VAN-HR	124.10 ± 2.10	152.38 ± 1.43	185.89 ± 3.63	126.72 ± 0.84	No-measurement*

*No-measurement in this work. However, an earlier GC-IRMS study [39] reports isotope delta value of −173.12 ± 2.2‰ or 128.79 ± 0.35 ppm (µmol/mol)

The median repeatability standard deviations of site-specific ^2^H/^1^H values in the range of 0.75%; 0.80%; 1.81%; and 0.57% for H1, H3, H4, and H5, respectively (Table S2) are very close to the critical points [41] and are sufficient for the comparison with the methoxy GC-IRMS, demonstrating reliability of ^2^H-qNMR measurements of vanillin. The results reported in Table 3 show that hydrogen isotope ratios at H1 and H5 show significant differences between the synthetic (VANA-1, VANB-1, VAN-1, VAN-4, VAN-8) and natural (VAN-K, VAN-HR) vanillin samples. Furthermore, the ^2^H/^1^H values for H1 differ significantly even among the synthetic vanillin samples, likely reflecting the variety of synthetic routes and/or starting materials for these vanillin samples [36, 49].

#### Comparison of PSIA GC-IRMS and ^2^H-qNMR results

**Fig. 3:**
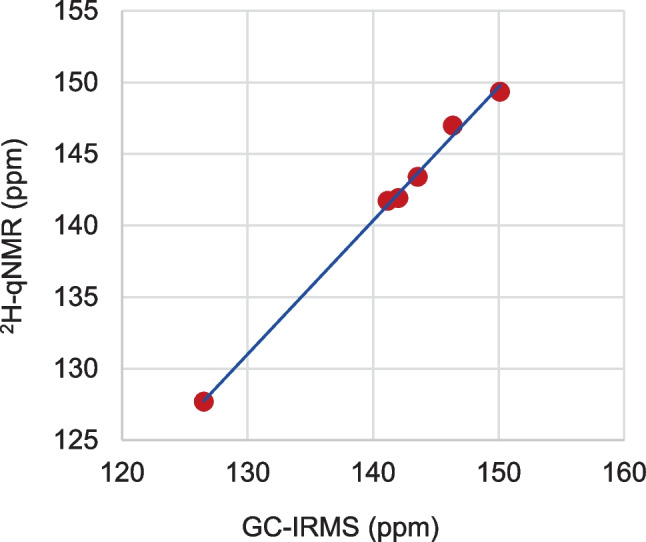
Comparison of hydrogen isotope of methoxy group (H5) measured by ^2^H-NMR and GC-IRMS. All values in ppm (µmol/mol)

Table 3 shows that the ^2^H-qNMR and GC-IRMS results for H5 all agree to within their uncertainties, with the absolute difference between the two methods being below 1 ppm (µmol/mol). Figure 3 shows remarkably consistent values of the (D/H)_5_ measurements in the methoxy group from six vanillin samples as measured by two independent methods. Regression line *y* = *bx*, where *b* = 0.9985 with standard uncertainty *u*(*b*) = 0.0020 (ordinary least squares), shows no significant bias between the two methods. Indeed, the largest difference between the two methods (1.19 ppm for VAN-K) is consistent with the combined measurement uncertainty of this difference (0.90 ppm) thus showing no significant difference between the two methods. This adds further demonstration to the reliability of both methods. It is also noteworthy to highlight the fact that both methods are traceable to entirely different reference materials: ^2^H-qNMR relies on TMU internal standard, whereas GC-IRMS uses two external standards traceable to VSMWO2. This comparison contributes to validate these independent isotopic measurements qNMR and GC-IRMS approaches.

### Measurement uncertainty

The standard uncertainty associated with the site-specific ^2^H/^1^H values obtained by ^2^H-qNMR measurements was evaluated by propagation of the individual uncertainty components that include the measurement reproducibility, uncertainty associated with the certified ^2^H/^1^H values of TMU and VSMOW, the molar masses of TMU and vanillin, as well as the chemical purity of vanillin. These calculation steps are documented in the supplementary information. Combining these sources of uncertainty yields standard uncertainty shown in Table 3.

### Effect of vanillin provenance on its isotopic composition

Bulk and site-specific hydrogen isotope ratios can be used to differentiate between various vanillin samples. Most synthetic vanillin is produced from the petrochemical precursor guaiacol [6, 8]. The isotope delta values for H5 for this synthetic vanillin range from −150‰ to +50‰ [15, 45, 46] (corresponding to isotope ratios from 132.4 to 163.6 ppm or µmol/mol), comparable to the values found in previous studies [35, 41]. The ^2^H/^1^H values of H5, obtained by GC-IRMS and ^2^H-qNMR, for VANA-1, VANB-1, VAN-1, VAN-4, and VAN-8 fall in this range. Furthermore, comparison of the ^2^H/^1^H isotope profile of these five vanillin materials to previous studies [35, 41] allows us to conclude that these are synthetic vanillin samples from guaiacol.

Natural vanillin samples have bulk isotope delta values ranging from −110‰ to −3‰ relative to VSMOW [15, 45, 46] and isotope ratios of H5 ranging from 127.5 to 123.5 ppm (µmol/mol) [16]. The site-specific isotopic composition of hydrogen obtained for vanillin VAN-HR (Table 3) and their bulk values (Table 1) correspond to vanillin from a vanilla pod. Furthermore, the carbon isotopic profile of VAN-HR (Table S3 in Electronic supplementary material) supports this hypothesis [21, 22]. A bulk value *δ*(^2^H) = −151.5‰ and isotope ratio *R*(^2^H/^1^H, H5) = 126.5 ppm (µmol/mol) for VAN-K (Tables 1 and 3), in addition to its carbon (Table S3) and hydrogen isotope profiles, suggest biosynthetic vanillin from ferulic acid (rice) [16, 32, 41, 45, 46]. Table 3 shows the ability of ^2^H-qNMR to differentiate between synthetic (VANA-1, VAN-1, VAN-1, VAN-4, VAN-8) and natural (VAN-K and VAN-HR) vanillin. As an example, hydrogen values of VANA-1 and VANB-1 are about the same for H3 and H5, but very different for H1, indicating a difference in the synthetic pathway.

## Conclusion

We have shown that the isotopic composition of hydrogen exhibits large variability among various vanillin samples. The variability of site-specific isotope ratios provides further ability to discern synthetic/biological synthetic histories of vanillin samples. Here, we show remarkably consistent measurements of ^2^H/^1^H of the vanillin methoxy group from six vanillin samples by two independent measurement methods, GC-IRMS and ^2^H-qNMR. GC-IRMS measurements provide three-fold smaller measurement uncertainties while also requiring considerably smaller sample sizes compared to ^2^H-qNMR. However, ^2^H-qNMR affords isotopic measurements of all hydrogen atoms (except for OH) of the vanillin molecule, while the GC-IRMS measures only the hydrogen atoms of the methoxy group via the appropriate chemical reaction. Previous studies have shown that ^13^C-qNMR measurements of ^13^C/^12^C isotope ratios of the methoxy group are consistent with those obtained using GC-IRMS [22] and Fig. S2 in Electronic supplementary material. This study extends such a comparison to the isotopic composition of hydrogen as a further contribution toward validation of ^2^H-qNMR [41] and GC-IRMS [16] methods for site-specific hydrogen isotope measurements. Thus, qNMR and GC-IRMS can be regarded as complementary analytical methods, and their combined use provides reliable results.

## Supplementary Information

Below is the link to the electronic supplementary material.

Supplementary file1 (DOCX 147 KB)

## Data Availability

The data supporting this study are available within the paper and its supplementary materials. Should any raw data files be needed in another format, they are available from the corresponding author upon reasonable request.
