# Supplementary material for: Site-specific hydrogen isotope measurements of vanillin by 2H-qNMR and GC-IRMS
Source: Anal Bioanal Chem. 2025 Jun 6;417(17):3987–96. doi: 10.1007/s00216-025-05920-1 (PMC12227362; doi:10.1007/s00216-025-05920-1)
Supplement: Supplementary file 1 — Supplementary file1 (DOCX 147 KB) [file 216_2025_5920_MOESM1_ESM.docx]

**Electronic Supplementary Material**

**Site-specific hydrogen isotope measurements of vanillin by ^2^H-qNMR and GC-IRMS**

Phuong Mai Le^1*^, Markus Greule^2^, Stanislav Sokolenko^3^, Serge Akoka^4^, Gérald Remaud^4^, Peter Costa^5^, Kathy Sharon Isaac^3^, Andre Simpson^5^, Frank Keppler^2^, and Juris Meija^1^

*^1^Metrology Research Centre, National Research Council Canada, 1200 Montreal Road, Ottawa, ON, K1A-0R6, Canada, ^2^Institute of Earth Sciences, Heidelberg University, Im Neuenheimer Feld 236, 69120 Heidelberg, Germany, ^3^Department of Process Engineering and Applied Science, Dalhousie University, 5273 DaCosta Row, Halifax, NS, B3H-4R2, Canada, ^4^CEISAM, Nantes Université-CNRS, UMR6230, 2 rue de la Houssinière, F-44000 Nantes, France, ^5^Environmental NMR Centre, University of Toronto, 1265 Military Trail, Toronto, ON, M1S-1A4, Canada*

*Corresponding author: Phuong Mai Le

[E-mail adresse : PhuongMai.Le@nrc-cnrc.gc.ca](mailto:*PhuongMai.Le@nrc-cnrc.gc.ca)

Keywords: Site-specific hydrogen isotope ratio, ^2^H-qNMR, GC-IRMS, vanillin authentication

**Table S1.** Quantities of vanillin and TMU (as an internal standard) and their information used for site-specific hydrogen isotope measurements of vanillin samples by ^2^H-qNMR

| **Samples** | **m_TMU_/g** | ***m*_VAN_/g** | **w_VAN_/(g/g)** | ***u*(w_VAN_)/(g/g)** |
| --- | --- | --- | --- | --- |
| **VANA-1** | 0.4326 | 2.1004 | 0.9962 | 0.0024 |
| **VANB-1** | 0.4514 | 2.0999 | 0.9954 | 0.0023 |
| **VAN-1** | 0.4195 | 2.0996 | 0.9856 | 0.0039 |
| **VAN-4** | 0.4537 | 2.1010 | 0.9931 | 0.0032 |
| **VAN-8** | 0.4464 | 2.1008 | 0.9932 | 0.0037 |
| **VAN-K** | 0.4322 | 2.0997 | 0.9695 | 0.0033 |
| **VAN-HR** | 0.2120 | 1.0501 | 0.9898 | 0.0030 |
| **Samples/standards** | Value | *u* | unit | note |
| *R*(^2^H/^1^H, VSMOW)  R(^2^H/^1^H, TMU) | 155.76  123.00 | 0.05  0.35 | ‰ vs VSMOW  ppm | Ref. [48]  ERM-AE003 |
| *M*_TMU_  *M*_VAN_ | 116.162  152.147 | 0.003  0.005 | g/mol  g/mol | C_5_H_12_N_2_O  C_8_H_8_O_3_ |

w is the chemical purity (mass fraction) of vanillin

M and m are the molecular weight and the weighted mass, respectively

R is the ^2^H/^1^H isotope ratio (ppm)

*u* is standard uncertainty

**Table S2.** Repeatability standard deviation of site-specific ^2^H/^1^H measurements by 2H-qNMR for vanillin samples. All values in %

| **Samples** | ***R*(H1)** | ***R*(H3)** | ***R*(H4)** | ***R*(H5)** |
| --- | --- | --- | --- | --- |
| **VANA-1** (n=4) | 0.40 | 1.26 | 1.68 | 0.64 |
| **VANB-1** (n=4) | 1.08 | 0.44 | 2.65 | 0.68 |
| **VAN-1** (n=3) | 1.48 | 0.24 | 0.83 | 0.49 |
| **VAN-4** (n=4) | 1.11 | 0.80 | 1.06 | 0.46 |
| **VAN-8** (n=4) | 0.75 | 1.13 | 1.86 | 0.57 |
| **VAN-K** (n=3) | 0.73 | 1.60 | 2.03 | 0.67 |
| **VAN-HR** (n=3) | 0.67 | 0.52 | 1.81 | 0.10 |
| **Median STDEV** | 0.75 | 0.80 | 1.81 | 0.57 |
| Critical point | ≤ 1.2 | ≤ 1.3 | ≤ 2.0 | ≤ 0.5 |

**Table S3.** Site-specific carbon isotope delta values, *δ*(^13^C), in vanillin samples measured by ^13^C-qNMR and GC-IRMS

with the associated 95% confidence intervals. All values reported in permilles (‰) relative to the VPDB.

| δ(^13^C) | VANA-1^[22]^ | VANB-1^[22]^ | VAN-1^[22]^ | VAN-4^[22]^ | VANA-8^[22]^ | VAN-K | VAN-HR |  |  |  |  |  |  |
| --- | --- | --- | --- | --- | --- | --- | --- | --- | --- | --- | --- | --- | --- |
| δ(^13^C_g_)  C_1_  C_2_  C_3_  C_4_  C_5_  C_6_  C_7_  C_8_-NMR  C_8_-IRMS | -31.30 ± 0.06  –21.36 ± 1.40  –32.09 ± 1.40  –32.52 ± 1.40  –28.26 ± 1.40  –29.84 ± 1.40  –29.38 ± 1.40  –23.73 ± 1.40  –53.25 ± 1.40  –53.53 ± 0.26 | -25.85 ± 0.05  -20.07 ± 1.40  –29.87 ± 1.40  –32.77 ± 1.40  –24.48 ± 1.40  –23.85 ± 1.40  –26.38 ± 1.40  –18.82 ± 1.40  –30.59 ± 1.40  –30.80 ± 0.16 | -29.81 ± 0.06  –22.81 ± 1.40  –32.94 ± 1.40  –33.24 ± 1.40  –31.64 ± 1.40  –23.31 ± 1.40  –27.53 ± 1.40  –21.04 ± 1.40  –46.53 ± 1.40  –46.76 ± 0.27 | -31.10 ± 0.08  –19.98 ± 1.40  –28.88 ± 1.40  –31.28 ± 1.40  –26.83 ± 1.40  –28.08 ± 1.40  –26.80 ± 1.40  –24.59 ± 1.40  –62.36 ± 1.40  –61.90 ± 0.29 | -26.19 ± 0.07  –22.03 ± 1.40  –29.92 ± 1.40  –33.59 ± 1.40  –28.96 ± 1.40  –19.02 ± 1.40  –24.02 ± 1.40  –18.32 ± 1.40  –33.20 ± 1.40  –33.81 ± 0.41 | -37.50 ± 0.03  –39.24 ± 1.40  –28.43 ± 1.40  –32.55 ± 1.40  –31.52 ± 1.40  –42.23 ± 1.40  –34.79 ± 1.40  –39.36 ± 1.40  –51.87 ± 1.40  –53.31 ± 0.41 | -19.96 ± 0.05  –13.26 ± 1.40  –19.44 ± 1.40  –18.03 ± 1.40  –20.16 ± 1.40  –27.51 ± 1.40  –19.85 ± 1.40  –23.73 ± 1.40  –18.01 ± 1.40  –19.03 ± 1.40 |  |  |  |  |  |  |

Determination of *T*_1_ values of ^2^H resonances on Bruker 500 MHz machine:

The *T*_1_ values of ^2^H resonances for vanillin sample were determined using the inverse recovery ‘t1ir’ and the ^2^H-VD list of 16 points in second: 0.001; 0.015; 0.025; 0.050; 0.100; 0.150; 0.200; 0.300; 0.400; 0.600; 0.800; 1.00; 1.25; 1.50; 3.00 and 6 s.

**Figure S1.** The fitting of ^2^H-NMR - T1 values for vanillin sample. The longest T1 of 0.688 s was observed at the peak 6 at 2.73 ppm - TMU (an IS).


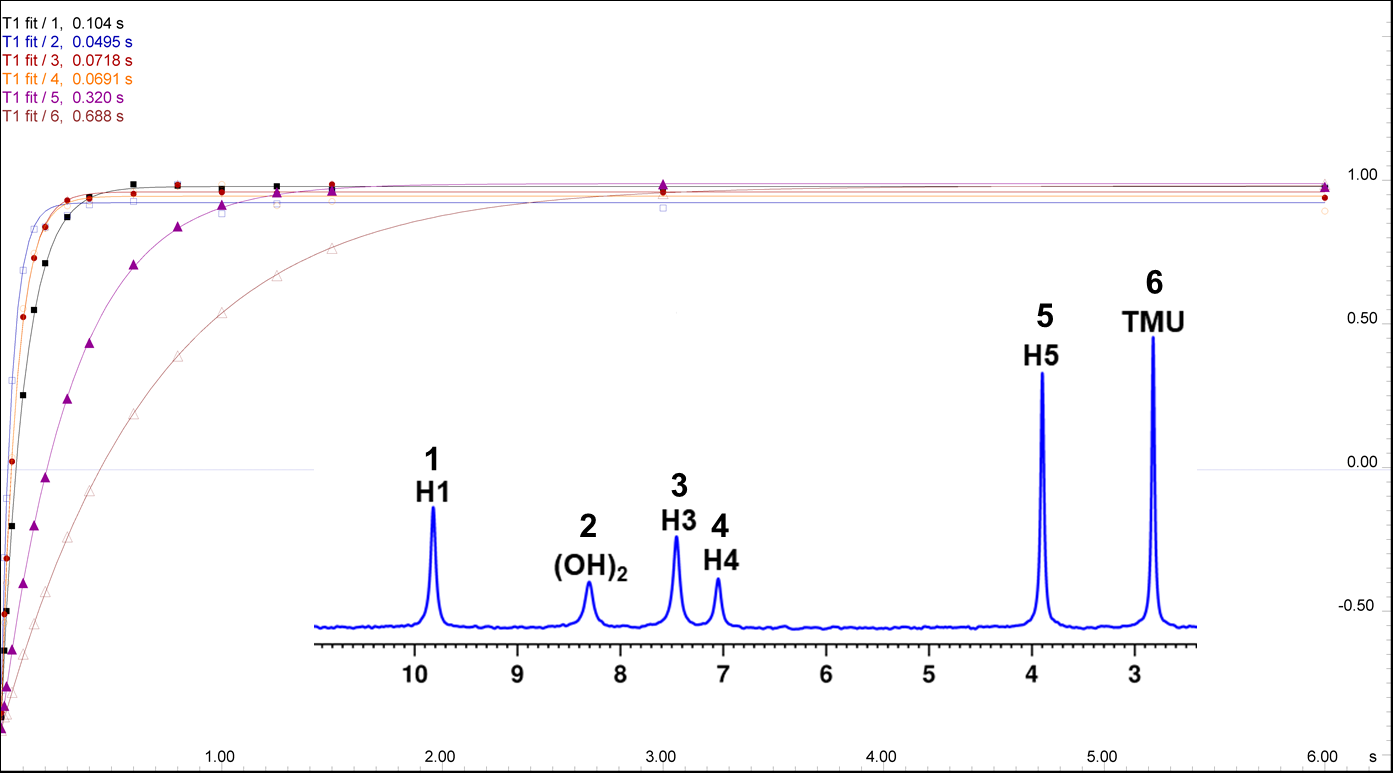


**Figure S2.** Interlaboratory comparison of ^13^C-qNMR and GC-IRMS methods for the carbon isotopic composition of vanillin methoxy group (C8). All values reported in permilles (‰) relative to the VPDB. Remarkably consistent results were obtained by these two methods for carbon isotope of methoxy group.

**References**

[22] Greule M, Le PM, Meija J, Mester Z, Keppler F. Comparison of carbon isotope ratio measurement of the vanillin methoxy group by GC-IRMS and ^13^C-qNMR. J Am Soc Mass Spectrom. 2024; 35:100-105.

**ACKNOWLEDGMENT**

PML would like to thank Michelle Chartrand, Yuliana Holowaty at NRC in Ottawa for their technical assistance for bulk δ^13^C experiments
